# Functional recovery after macula involving retinal detachment and its correlation with preoperative biomarkers in optical coherence tomography

**DOI:** 10.1007/s00417-021-05113-3

**Published:** 2021-03-06

**Authors:** Julian E. Klaas, Philip Rechl, Nikolaus Feucht, Jakob Siedlecki, Julia Friedrich, Chris P. Lohmann, Mathias Maier

**Affiliations:** 1 Klinik und Poliklinik für Augenheilkunde, Technische Universität München, Munich, Germany; 2 Klinik für Augenheilkunde, Universitätsklinikum Freiburg, Freiburg im Breisgau, Germany; 3 Augenklinik der Universität München, Ludwig-Maximilians-Universität, Munich, Germany

**Keywords:** MIRD, CIRD, Macula Involving Retinal Detachment, Center Involving Retinal, Detachment, Macula-off, Rhegmatogenous, Macula-on, Fovea-off, OCT, Optical coherence tomography, Morphology, Retinal detachment, Biomarker, Nomenclature

## Abstract

To introduce an ETDRS grid-based classification for macula involving retinal detachment (MIRD) with or without center (foveal) involvement and to identify biomarkers in preoperative optical coherence tomography (OCT) associated with a favorable postoperative functional outcome in eyes with center involving retinal detachment (CIRD). One hundred and two eyes of 102 consecutive patients (f/m: 35/67) with primary rhegmatogenous retinal detachment, preoperative evidence of MIRD (perifoveal involvement of ≤ 6.0 mm), and successful retinal surgery were included in this retrospective cohort study. Eyes were assigned to 5 grades of MIRD (G1–G5), based on the extent of detachment in the ETDRS grid. Eyes with a detached foveal status (CIRD) were assigned to G4 or G5. In CIRD, the following OCT biomarkers were quantified and correlated with mean BCVA (logMAR) at 3 months postsurgery, using univariate and multivariable regression models: grade of detachment, extent of intraretinal edema, height of foveal detachment, subretinal folds, and epiretinal membrane. Forty-one of 102 eyes (40.2%) presented with an attached foveal status, defined as either outer (G1: 11.8%) or inner (G2: 18.6%) macular involvement or fovea-threatening MIRD (G3: 9.8%). Sixty-one eyes (59.8%) showed CIRD (G4 or G5). Eyes with CIRD had significantly worse postoperative BCVA than eyes without foveal involvement (0.355 logMAR vs. 0.138 logMAR, *p*<0.001). If CIRD was limited to three outer ETDRS quadrants (G4), mean BCVA was better compared to CIRD involving all four ETDRS quadrants (G5) (0.254 logMAR vs. 0.522 logMAR, *p*<0.001). Reading ability (BCVA ≤ 0.4 logMAR) was restored in 97.6% of eyes with G1–G3 compared to 86.9% of eyes with G4 (*p*=0.072) and 52.4% of eyes with G5 (*p*<0.001). In multivariable regression analysis of eyes with CIRD, a lower grade of detachment (G4 vs. G5: *p*<0.05) and lower extent of cystoid edema (focal/none vs. wide: *p*<0.001) were both associated with better postoperative function. The functional outcome after MIRD may be worse in the presence of foveal involvement (CIRD), but a lower grade of detachment and the absence of intraretinal edema can predict a good recovery in spite of CIRD.

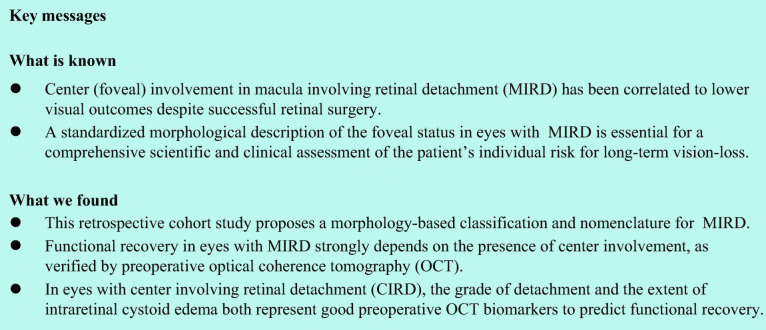



## Introduction

Rhegmatogenous retinal detachment (RRD) is a sight-threatening condition that requires urgent surgical treatment. Even though rates of successful retinal reattachment have continuously risen over the past decades, visual outcomes are difficult to predict and vary widely [2–8]. Factors associated with worse functional and anatomical results after successful retinal surgery involve individual risk factors (e.g., duration of symptoms, age, high myopia, and concomitant retinal disease), peri- and postoperative factors (time to surgery, type of surgery, day of the week, experience of surgeon, positioning before and after surgery, and follow-up management), and anatomical features on presentation (e.g., foveolar involvement and hole localization) [9–12]. Furthermore, differences in functional outcomes and subsequent functional recovery have been explained by morphology-function correlations using postoperative optical coherence tomography (OCT) examination [8, 13–17]. Even though a detached foveal status is widely considered to be prognostic of slower and overall reduced functional recovery, full rehabilitation of visual acuity seems possible in many cases. Several studies have tried to identify prognostic factors that could predict a preferable functional outcome in the presence of center (foveal) involvement, but few reports have used preoperative SD-OCT imaging to describe and classify the morphological extent of macular and center involvement in a detailed and standardized manner [11, 18–20]. In view of the consequential inconsistencies and possibly low comparability between available reports, the purpose of this retrospective study was

1. to introduce an ETDRS grid-based classification system for the macular status in eyes with macula involving retinal detachment (MIRD)
2. to identify morphological criteria (biomarkers) in preoperative OCT scans which correlate to a favorable postoperative functional recovery after center involving retinal detachment (CIRD)

## Methods

This study reports a retrospective review of one hundred and two eyes of 102 consecutive patients who underwent successful surgical treatment for primary RRD at the Department of Ophthalmology of Technical University Munich (TUM), Germany. This study was performed in consensus with the Declaration of Helsinki. It was reviewed and admitted by the institutional review board and ethics committee of TUM.

### Data acquisition

**Fig. 1:**
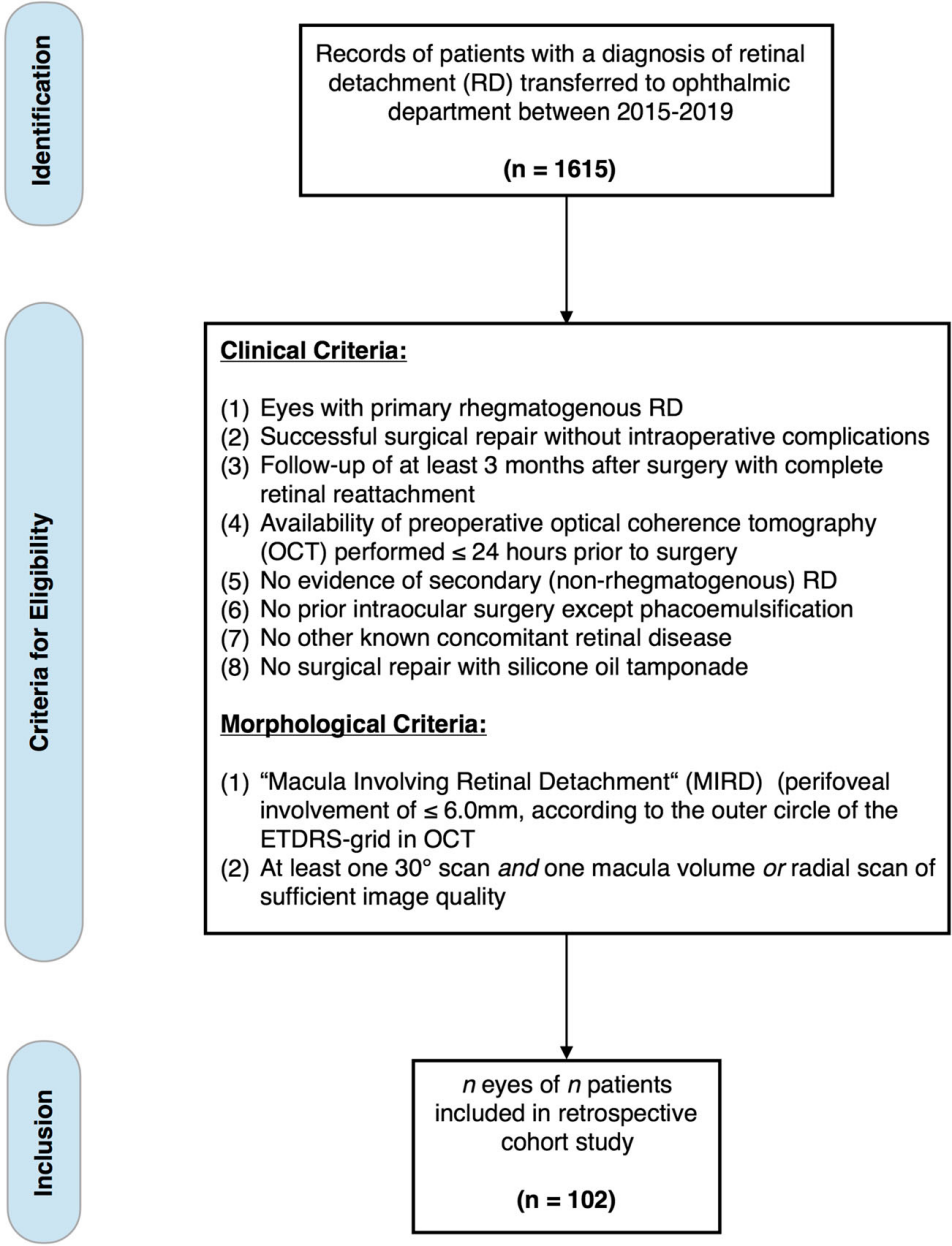
Flow chart depicting process of data acquisition and eligibility criteria for in- and exclusion of patients resulting in a retrospective cohort of 102 eyes of 102 patients with primary rhegmatogenous macula involving retinal detachment (MIRD)

The clinical and morphological data of 1615 patients transferred to the ophthalmic department between January 2015 and January 2019 with a diagnosis of RD were reviewed for the following inclusion and exclusion criteria (Fig. 1). Inclusion criteria: (1) primary RRD, (2) successful surgical repair without intraoperative complications, (3) follow-up of at least 3 months after surgery with complete reattachment, and (4) availability of preoperative OCT performed ≤ 24 hours before surgery (at least one 30° scan *and* one full volume (or radial) scan). Exclusion criteria: (1) prior intraocular surgery except phacoemulsification, (2) vitreoretinal surgery with silicone oil tamponade, (3) concomitant retinal disease, and (4) evidence of secondary (nonrhegmatogenous) RD, such as traumatic, PVR-related, diabetic, uveitic, or exsudative RD. If forementioned criteria were met, morphological data of preoperative OCT were subsequently screened (Software: Heyex 2, Heidelberg Engineering, Germany) for the presence of “macula involving retinal detachment” (MIRD), a term we defined as perifoveal involvement of ≤ 6.0 mm, according to the outer circle of the ETDRS grid in OCT, resulting in one hundred and two eyes of 102 patients fulfilling all aforementioned clinical and morphological criteria (Fig. 1).

### Participants and clinical setting

Eligible patients received immediate and comprehensive ophthalmic examination, including BCVA assessment (decimal), biomicroscopic and funduscopic exam through a dilated pupil of both eyes, and 30° spectral domain-OCT (SD-OCT) before surgery. Symptoms at presentation included sudden or progressive perception of “mouches volantes,”areal shadows, temporal photopsia, or a subjective decline in visual function. Diagnosis of RRD was based on both clinical examination and imaging results assessed by at least one resident and one senior ophthalmic surgeon. Surgery was not delayed more than 1 day after admission and was performed by four different experienced retinal surgeons (surgical experience > 10 years). Apart from regular follow-up examinations, patients had been recommended to arrange follow-up visits at 3 months after surgery. At this follow-up, SD-OCT examination was conducted to record postsurgical cystoid macular edema and to monitor regeneration of outer retinal layers.

### Morphological review and classification

**Fig. 2:**
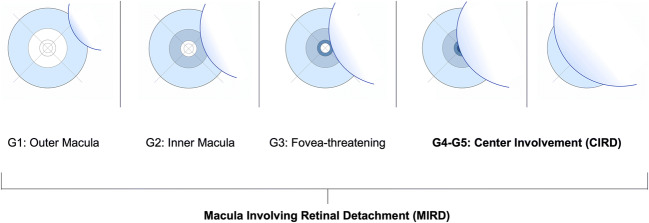
Illustration for 5 grades of macula involving retinal detachment (MIRD) based on the morphological extent of involvement using the ETDRS grid in optical coherence tomography (OCT)

**Fig. 3:**
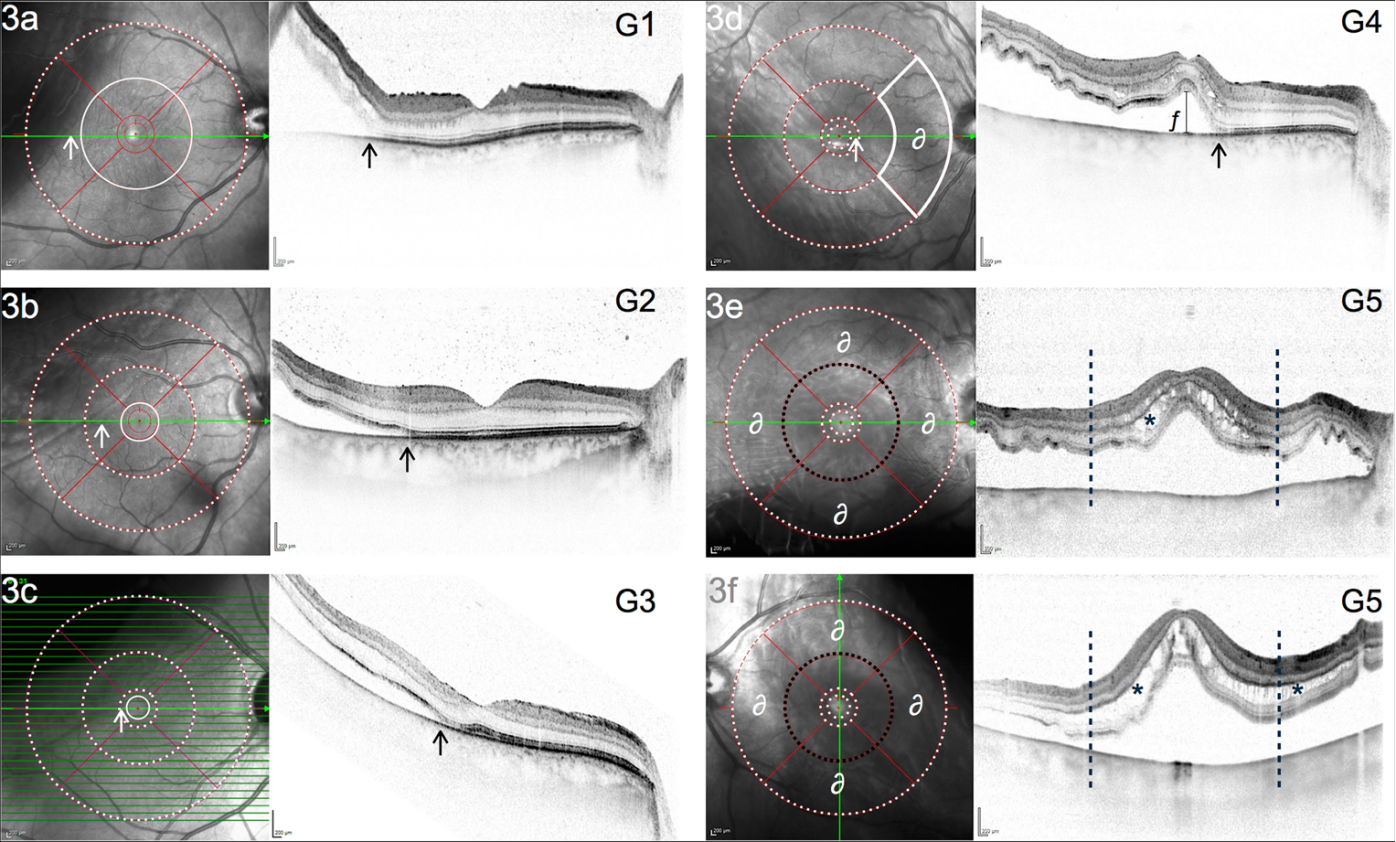
Infrared-image (IR) with ETRDS grid and SD-OCT (30°) of 6 eyes, representing 5 grades of macula involving retinal detachment (MIRD). a–c represent MIRD without center involvement (G1–G3). d CIRD with limited macular detachment (G4): ∂ marks one outer macular quadrant where no subretinal fluid can be detected in volume or radial scans. e, f show detachment involving all 4 outer macular quadrants (marked with ∂) defined as G5 (“CIRD with complete macular detachment“). Dotted white circles in a–c indicate subretinal fluid within this circle. A drawn through white circle indicates that subretinal fluid does not cross this boundary. White and black arrows mark margin of detachment correlating to localization in IR/OCT scans; ƒ in d represents height of foveal detachment (FDH = 291 μm, measured perpendicularly to RPE in 1:1 resolution); * marks focal (e) and wide (f) cystoid intraretinal edema with a perifoveal radius of ≤ 3.0 mm (black dotted circle/lines) serving as cutoff

Morphological data of preoperative SD-OCT-scans were screened and reviewed independently by 2 experienced investigators (residents). A senior investigator was consulted to confirm the final agreement if no intergrader agreement could be established. Patients with MIRD were classified into 5 subgroups, depending on their preoperative macular status as follows: The extent of macular involvement was assessed using the ETDRS grid, available as an overlay tool in a variety of imaging systems. In this study, we used the ETDRS grid provided by Heyex 2 Software (Heidelberg Engineering, Heidelberg, Germany). The ETDRS overlay was carefully centered on the fovea with Infrared-Reflection (IR) and B-Scan image serving as morphological correlates. Typical microstructural and anatomical features were used to locate the fovea, such as the location of the foveal contour in B-Scan images and topographical relation to retinal vasculature and optic nerve head in IR images. Figure 2 illustrates the 5 advancing grades (G) of this proposed classification system, as applied to all patients reviewed in this study. Grades 1–3 (G1–G3) are distinguished by an attached foveal status, in contrast to a detached fovea (“center involvement”) in grades 4 and 5 (G4–G5). G4 describes CIRD as limited to three outer quadrants of the ETDRS grid in comparison to G5, which is characterized by a complete macular detachment, involving all 4 outer quadrants (Fig. 3). In terms of nomenclature, we followed the zonal (anatomical) naming of the ETDRS grid while also considering the clinical practicability of the following labels: G1: “MIRD with outer macular involvement,” G2: “MIRD with inner macular involvement,” G3: “fovea-threatening MIRD,” G4: “CIRD with limited macular detachment,” and G5: “CIRD with complete macular detachment” (Fig. 2). All available scans of patients with CIRD were examined for the following biomarkers: intraretinal edema (IE), subretinal folds (SF), epiretinal membrane (ERM), and height of foveal detachment (FDH). At least one 30° scan through the fovea and one full volume or radial scan were available in all patients. IE was quantified as either none or focal or wide edema. Focal edema was defined as not extending beyond the 3.0 mm mark of the ETDRS grid. Wide IE was defined as a (peri-)foveal cystoid edema with continuous intraretinal fluid of more than 3.0 mm radial extension into any peripheral direction (Fig. 3e+3f). The amount of edema was measured by two investigators independently. All available 30° and volume/radial scans for each patient were reviewed using the ETDRS grid to distinguish between focal or wide edema. Since no agreement was found in 4 of 61 cases, a senior investigator was consulted, which led to the successful quantification of 59 of 61 eyes (96.72%). SF was counted as “present,” when more than 4 folds were visible on the central foveal scan and when folds were higher than their base. FDH was measured (1:1 μm scale) subfoveally and perpendicularly to retinal pigment epithelium (RPE) (Fig. 3d). If different values were measured by the two researchers, a mean value was calculated after errors of measurement techniques had been excluded. If FDH could not be measured because of complete macular detachments with high amounts of subretinal fluid, a value of 1200 μm was used for statistical analysis, since the highest measurable value in our collective was 1153 μm. ERM was documented as present vs. absent without quantification. Due to overall varying assessments of external limiting membrane (ELM) and ellipsoid zone (EZ) integrity, we did not evaluate for the reflectance of outer retinal layers preoperatively. However, the integrity of EZ was examined on *postoperative* OCT (if available) and subdivided into three groups: intact EZ, irregularities of EZ reflectance, and complete interruption of EZ.

### Main outcomes and measures

The main outcome measure was mean postoperative BCVA (logMAR) and a favorable functional outcome, defined as BCVA ≤ 0.4 (logMAR) and subsequently referred to as “reading ability.” The secondary outcome measure was postoperative morphology. The main outcome measure and secondary outcome measure were both correlated with morphological features in preoperative OCT.

### Statistical analysis

Statistical analysis was performed with IBM® SPSS® Statistics (Version 26.0.0.0) in coordination with the department of statistics of TUM. For statistical analysis, BCVA values (decimal) were transformed into their logMAR equivalent. Nondecimal values such as perception of hand movement (HM) and counting fingers (CF) were replaced as follows: HM = 2.2 , CF = 1.9 [21]. The mean postoperative BCVA was correlated as a continuous variable with preoperative clinical factors using parametric tests for 2 or more independent groups (*t*-test, ANOVA) and simple linear regression analysis models for continuous preoperative variables. Variance homogeneity was tested with the Levene test. Normal distribution was assumed for a sample size of ≥ 25 and by individual inspection of distribution graphs. In the case of extreme skewness, outliers were removed after careful evaluation of their effect. A multivariable linear regression model was performed observing parameters found to correlate significantly in prior univariate regression analysis. A 2-sided *p*-value of 0.05 was used for all tests.

## Results

One hundred and two eyes of 102 patients were included (female = 35, male = 67; mean age = 62.80, SD 12.34). Mean preoperative BCVA was 0.742 (SD 0.737) logMAR. Mean postoperative BCVA was 0.268 (SD 0.269) logMAR. Surgical methods comprised vitrectomy and scleral buckling surgery as well as combined procedures. Univariate regression analysis showed significant correlations with postoperative BCVA for the following clinical baseline data: preoperative BCVA (*p*<0.001, ß = 0.487) and type of surgery (*p*<0.05). With respect to the latter, vitrectomy (±phaco) with encircling band was associated with a worse postoperative functional outcome compared to vitrectomy (±phaco) (*p*=0.002) or buckling procedures alone (*p*<0.001) (Table 1)*.* In the following, we report the findings of our main (Section 1–5) and secondary (Section 6) outcome measures: Sections 1–4 document the results of univariate linear regression models for each defined biomarker in preoperative OCT. Section 5 provides the results of multiple linear regression analysis. Section 6 discusses associations between pre- and postoperative morphology. Table 1 summarizes demographic, clinical, and morphological baseline characteristics and the results of univariate and multiple regression analysis models.

**Table 1:** Demographic, clinical, and morphological baseline data in correlation to postoperative BCVA

Baseline data of study cohort	Total	Mean BCVA 3 m follow-up	Correlation with mean BCVA 3 m follow-up			
Category	no (%) or mean (SD)	in logMAR (SD)	Univariate linear regression		Multiple linear regression	
			*B* (SE)	*p* value	*B* (SE)	*p* value
Gender	102 (100)	0.268 (0.269)	–	0.567		
Male	67 (65.68)	0.257 (0.280)	Reference	Reference	–	–
Female	35 (34.31)	0.289 (0.248)	0.032 (0.056)	0.567	–	–
Age (years)	62.80 (12.343)	0.268 (0.269)	0.002 (0.002)	0.279	–	–
Lens status^†^	102 (100)	0.268 (0.269)	–	0.811	–	–
Phakic	52 (50.98)	0.259 (0.273)	Reference	Reference	–	–
Pseudophakic	50 (49.02)	0.272 (0.269)	0.014 (0.057)	0.811	–	–
Eye (side)	102 (100)	0.268 (0.269)	–	0.992	–	–
Right	59 (57.84)	0.267 (0.284)	Reference	Reference	–	–
Left	43 (42.16)	0.268 (0.249)	0.001 (0.054)	0.992	–	–
BCVA (on admission), logMAR	0.742 (0.737)	0.268 (0.269)	0.178 (0.032)	0.000^a^	0.002 (0.072)	0.976
Duration of symptoms (days)^∑^	7.52 (8.373)	0.263 (0.268)	0.002 (0.003)	0.415	–	–
Surgical method	102 (100)	0.268 (0.269)	–	0.020	–	0.000
Vitrectomy	42 (41.18)	0.230 (0.212)	0.069 (0.079)	0.381	0.128 (0.080)	0.116
Vitrectomy (+ encircling band)	19 (18.63)	0.448 (0.410)	0.287 (0.090)	0.002	0.506 (0.096)	0.000
Phacovitrectomy	13 (12.75)	0.261 (0.231)	0.100 (0.098)	0.310	0.078 (0.105)	0.460
Phacovitrectomy (+encircling band)	6 (5.88)	0.387 (0.253)	0.225 (0.125)	0.073	0.029 (0.120)	0.813
Scleral buckling	8 (7.84)	0.145 (0.147)	–0.016 (0.113)	0.888	–0.113 (0.130)	0.387
Scleral buckling (+ p. retinopexy)	14 (13.73)	0.161 (0.142)	Reference	Reference	Reference	Reference
Morphological data (OCT biomarker)						
Extent of macular involvement	102 (100)	0.268 (0.269)	–	0.000	–	0.034^¶^
G1	12 (11.76)	0.132 (0.129)	–0.390 (0.082)	0.000	–	–
G2	19 (18.62)	0.160 (0.174)	–0.362 (0.071)	0.000	–	–
G3	10 (9.80)	0.106 (0.092)	–0.416 (0.087)	0.000	–	–
G4	38 (37.25)	0.254 (0.330)	–0.268 (0.061)	0.000	–0.159 (0.073)	0.034
G5	23 (22.55)	0.522 (0.279)	Reference	Reference	Reference	Reference
Foveal detachment height^∆^	692.39 (381.29)	0.355 (0.298)	0.000 (0.000)	0.004^b^	–0.000 (0.000)	0.760
Intraretinal edema^∆¥^	59 (100)	0.351 (0.299)	–	0.004	–	0.001
None	12 (20.34)	0.226 (0.265)	–0.284 (0.090)	0.003	–0.182 (0.076)	0.021
Focal (perifoveal radius ≤ 3.0 mm)	19 (32.20)	0.196 (0.144)	–0.314 (0.078)	0.000	–0.314 (0.080)	0.000
Wide (perifoveal radius > 3.0 mm)	28 (47.46)	0.510 (0.316)	Reference	Reference	Reference	Reference
Subretinal folds^∆^	61 (100)	0.355 (0.298)	0.141 (0.086)	0.109	–	–
Epiretinal membrane^∆^	61 (100)	0.351 (0.299)	0.235 (0.117)	0.050	–	–

*no* number, *m* months, *SD* standard deviation, *B* unstandardized coefficient, *ß* standardized coefficient, *SE* standard error, *p.* pneumatic. ^¶^Multiple linear regression model considering clinical and morphological variables found significant in univariate analysis; *R* squared = 0.637; adjusted *R* squared = 0.562. ^a^2-tailed significance at 0.01 level (Pearson correlation = 0.487). ^b^2-tailed significance at 0.01 level (Pearson correlation = 0.361). ^†^Results of statistical correlation with postoperative BCVA (3 months after surgery) were calculated with postoperative lens status data; mean BCVA at 3-month follow-up refers to follow-up lens status. ^∑^Data for duration of symptoms was only available for 97 patients. ^∆^Parameter was measured only in eyes with center involving retinal detachment (61 eyes with CIRD). ^¥^The quantification of intraretinal edema was possible in 59 of 61 eyes

### Prognostic value of MIRD classification

102 eyes were assigned to 5 different grades of macular involvement as illustrated in Figs. 2 and 3. In total, 61 of 102 eyes showed foveal involvement (CIRD) and were subsequently assigned to G4 (*n*=38) or G5 (*n*=23). 41 eyes were assigned to G1–G3 since no foveal involvement was present. Mean BCVA in grades *with* center involvement was 0.254 logMAR (SD 0.330) for G4 and 0.522 logMAR (SD 0.279) for G5 (see Table 1). Mean BCVA in grades without center involvement was as follows: G1: 0.132 (SD 0.129); G2: 0.160 (SD 0.174); G3: 0.106 (SD 0.092). Thus, eyes with CIRD had a significantly worse functional outcome than eyes with an attached fovea (*p*<0.001). When only eyes with G4 were considered, this was also true: worse postoperative BCVA values were found in eyes with center involvement and *limited* macular detachment (G4) compared to eyes without foveal involvement (G1–G3) (*p*=0.008). Eyes with G5 in turn showed worse functional outcomes than eyes with G4 (*p*<0.001, *ß* = 0.439). There was no statistical difference in mean BCVA values (logMAR) between grades G1–G3. Reading ability (BCVA≤ 0.4 logMAR) was restored in 40 of 41 patients (97.6%) with G1–G3 in comparison to 33 of 38 patients (86.8%) with G4 (*p*=0.072) and only 12 of 23 patients (52.3%) with G5 (*p*<0.001). In summary, a favorable outcome, such as reading ability, was reached very frequently in grades G1–G4, whereas this outcome was significantly less frequent in eyes with G5 (*p*<001). In summary, center involvement correlated with worse functional outcomes, but the extent of center involvement as assessed by the ETDRS grid (G4 vs. G5) had a significant impact on functional recovery at 3 months after surgery (see Table 2).

**Table 2:** Functional outcome after CIRD in correlation with preoperative extent of intraretinal edema

Extent of macular detachment in CIRD	Total	Quantification of intraretinal edema		
		None	Focal (≤ 3.0 mm)^a^	Wide (> 3.0 mm)^a^
CIRD with *limited* macular detachment (G4)				
Number of patients in subgroup, *n* (%)	38 (100)	10 (100)	16 (100)	12 (100)
Number of patients with BCVA ≤ 0.4 logMAR^*^, *n* (%)	33 (86.84)	10 (100)	15 (93.8)	8 (66.67)
Mean BCVA in logMAR (SD)	0.254 (0.227)	0.141 (0.103)	0.200 (0.147)	0.419 (0.227)
*p* value^b^	–	0.003	0.007	Reference
*B*^b^	–	–0.278 (SE 0.086)	–0.219 (SE 0.076)	Reference
CIRD with *complete* macular detachment (G5)				
Number of patients in subgroup, *n* (%)	21 (100)	2 (100)	3 (100)	16 (100)
Number of patients with BCVA ≤ 0.4 logMAR^*^, *n* (%)	11 (52.38)	1 (50)	3 (100)	7 (43.75)
Mean BCVA in logMAR (SD)	0.528 (0.337)	0.650 (0.494)	0.174 (0.156)	0.579 (0.319)
*p* value^b^	–	0.766	0.059	Reference
*B*^b^	–	0.072 (SE 0.239)	–0.404 (SE 0.200)	Reference
CIRD with limited *or* complete detachment (G4+G5)^c^				
Number of patients in subgroup, *n* (%)	59 (100)	12 (100)	19 (100)	28 (100)
Number of patients with BCVA ≤ 0.4 logMAR^*^, *n* (%)	44 (74.58)	11 (91.67)	18 (94.73)	15 (53.57)
Mean BCVA in logMAR (SD)	0.351 (0.299)	0.226 (0.265)	0.196 (0.144)	0.510 (0.316)
*p* value^b^	–	0.003	0.000	Reference
*B*^b^	–	–0.284 (SE 0.090)	–0.314 (SE 0.078)	Reference

^*^BCVA ≤ 0.4 logMAR (= BCVA ≥ 0.4 decimal) is referred to as “reading ability.” ^a^Perifoveal radius, according to the 3.0 mm boundary of the ETDRS grid. ^b^Univariate analysis of variance (ANOVA): dependent variable: mean BCVA (logMAR); independent variable: extent of intraretinal edema: *none* vs. focal vs. *wide*. ^c^*R* squared = 0.261; adjusted *R* squared = 0.234. *n* number, *SD* standard deviation, *SE* standard error, *B* unstandardized coefficient

### Intraretinal edema in eyes with CIRD

The extent of preoperative intraretinal edema was successfully quantified in 59 of 61 eyes with CIRD. Quantification could not be performed accurately in 2 cases due to lower OCT image quality. The following data for preoperative evidence of intraretinal cysts were found (Table 2): 28 patients had wide cystoid edema, 19 patients had focal edema, and 12 patients showed no perifoveal cysts. Mean BCVA (logMAR) between groups with focal (0.196, SD 0.144) and no edema (0.226, SD 0.265) was similar and of no significance (*p*=0.114). However, eyes with preoperative evidence of *wide* intraretinal edema showed worse BCVA (0.510, SD 0.316) compared to eyes with focal (*p*<0.001, *B* = -0.314) or no edema (*p*=0.003, *B* = - 0.284). This relationship was also significant with regard to reading ability: 91.7% of eyes with no intraretinal cysts and 94.7% of eyes with focal cystoid edema reached reading ability compared to only 53.6% of cases with wide cystoid edema (*p*<0.001). Since there was a higher proportion of eyes with wide edema within G5 (76.2%) compared to G4 (31.6%), additional analysis was performed for G4 only, in order to examine fluid-dependent functional differences for this subgroup with a more balanced sample size. In this respect, similar results could be reproduced: In G4, mean BCVA (logMAR) was significantly worse in the presence of wide edema (0.419, SD 0.227) compared to focal (0.141, SD 0.103, *p*=0.007) or no edema (0.200, SD 0.147, *p*=0.003). Reading ability was reached in 25 of 26 cases (96.2%) if there was either no or focal cystoid edema, in comparison to only 66.7% of cases with wide edema (*p*<0.05). Of note, if there was a complete absence of intraretinal cysts, reading ability was reached in 100% of cases with G4. With regard to disease duration, wide intraretinal edema was slightly more common in patients, who had symptoms ≥ 7 days in comparison to patients with symptoms <7 days (55.0% vs. 40.5%, respectively), but overall, no significant correlation between duration of symptoms and extent of intraretinal edema was found in our collective.

### Foveal detachment height in eyes with CIRD

Univariate linear regression analysis revealed a significant inverse correlation between postoperative visual function and height of foveal detachment (Table 1): Higher detachments were associated with a lower visual acuity at 3 months after surgery (*p*<0.01 , *ß* = 0.361). This correlation lost its statistical significance for FDH values ≥ 445 μm (*p* ≥ 0.05). Thus, a cutoff of 500 μm was empirically selected to compare functional results between eyes above and below this value. A high significance for this cutoff with regard to morphology-function correlation was found: Eyes with a detachment height of ≥ 500 μm had a significantly worse mean BCVA (0.236, SD 0.215) than eyes with FDH < 500 μm (0.438, SD 0.321, *p*=0.005). Furthermore, lower measurements of subfoveal fluid were significantly correlated with preoperative evidence of no/focal edema, less than 4 subretinal folds, or a G4 detachment, respectively.

### Subretinal folds and epiretinal membrane in eyes with CIRD

Mean BCVA (logMAR) was worse in patients with preoperative evidence of ERM (0.559, SD 0.348) in comparison to eyes without ERM (0.323, SD 0.284), but no significant relationship could be established (*p* = 0.05). A similar relationship was observed for preoperative evidence of ≥ 4 subretinal folds, though this was not significant either (*p* = 0.109).

### Multivariable linear regression model in eyes with CIRD

A multivariable linear regression model of all preoperative factors that were found significant in prior univariate analysis for eyes with CIRD showed remaining significance for type of surgery (*p*<0.001), extent of macular detachment (*p*<0.05), and extent of intraretinal edema (*p*=0.001). No significant correlation with postoperative outcome could be established for preoperative BCVA and foveal detachment height. Table 1 illustrates the changes of raw coefficients (*B*) between univariate and multiple regression analysis models. This multivariable model could explain up to 63.7% of the variance in postoperative BCVA (*R*^2^ = 0.637).

### Postoperative morphology in correlation with preoperative OCT biomarkers

As a secondary outcome measure, we correlated *postoperative* morphology (e.g., evidence of intraretinal fluid, EZ integrity) with preoperative biomarkers in OCT and postoperative BCVA. As expected, eyes with no or minor EZ irregularities at 3 months after surgery were associated with a better BCVA compared to eyes with a complete disruption in EZ reflectivity (*p*=0.004 and *p*=0.022, respectively). With regard to preoperative biomarkers in OCT, a significant correlation between the preoperative extent of intraretinal edema and postoperative evidence of EZ defects was found (Fisher’s exact test, *p* = 0.01): If there was a preoperative *absence* of wide intraretinal edema, postoperative EZ evaluation *never* revealed any major defects in the EZ layer at 3 months after surgery. By contrast, 25.9% of patients *with* preoperative evidence of wide edema later showed a major disruption in EZ reflectivity. Of note, all patients with a major disruption of EZ integrity at 3 months after surgery had shown evidence of wide intraretinal edema prior to surgical intervention. However, no statistical relationship between the amount of preoperative and postoperative intraretinal fluid was found.

## Discussion

With this retrospective study, we have provided insight into a morphological classification for macula involving retinal detachment (MIRD) with outer macular (G1) or inner macular involvement (G2), threatened foveal status (G3), or center involvement (CIRD: G4+G5).

Center involvement has long been associated with slower and worse functional recovery, even after prompt retinal surgery [18, 22–24]. Our study could reproduce this assumption: Overall, eyes with foveal involvement (CIRD) had a significantly worse visual function at 3 months after surgery compared to eyes with no foveal involvement (G1–G3). However, significant functional differences *between* eyes with CIRD were documented in our study. We were able to relate these varying functional results to a close interaction of preoperative OCT biomarkers: A *limited* extent of macular detachment (G4) and the absence of wide intraretinal edema were strongly associated with a favorable functional outcome despite a detached foveal status. While both grades of CIRD (G4 and G5) were generally associated with a significant drop in mean postoperative BCVA, a respectable rate of patients with G4 (86.9%) showed restoration of reading ability (BCVA ≤0.4 logMAR) at only 3 months after surgery, despite prior center involvement and its frequently assumed association with a worse or slower visual recovery. The high significance of overall *worse* BCVA values in G4 (compared to G1–G3) but not in terms of reading ability hints to the possibility that eyes with G4 (and few intraretinal cysts) might in fact have a respectable if not in some cases even excellent long-term outcome, albeit a possibly *slower* recovery when compared to G1–G3. Studies, which have shown, that EZ restoration in eyes with *or* without foveal involvement tends to occur to a significant amount in both groups (“fovea-off” vs. “fovea-on”) even at 12 months after surgery, support this assumption [15, 25, 26]. Nevertheless, we cannot know if the differences in visual recovery found in this study would approximate each other after a longer follow-up time in eyes with G4 vs. G1–G3. Given the highly significant drop in both absolute BCVA values *and* reading ability of eyes with G5, it seems unlikely that the same assumption might be true for these cases as well. But even though a high proportion of cases with G4 indeed seems to experience a beneficial functional recovery, the surgical immediacy of different grades of MIRD should be critically discussed on the basis of our proposed classification and future studies, especially with regard to the subtle and delicate morphological transition from a fovea-threatening (G3) to a detached foveal status (G4). While appropriate positioning of the patient until time of surgery would be recommended independently from any estimate given here, it is not well documented in how much time a G1 or G2 status would progress to a threatened or even center involving grade [27, 28]. For instance, we found one patient who had been admitted with MIRD G3 in the evening prior to surgery. On admission, OCT had shown no intraretinal cysts. The OCT immediately before surgery (less than 24 hours after first OCT) already revealed a G4 situation with widespread intraretinal edema in spite of correct positioning and overnight admission to our clinic. Apart from the possible prognostic drop in the functional outcome which might have been evaded by earlier surgery, in this case, another notable finding can be observed here: the preoperative evidence of intraretinal fluid may not always be associated with a longer duration of retinal detachment. Remarkably, in the present study, we could only document a weak association between the preoperative extent of intraretinal edema and a longer duration of symptoms (≥7 days). Cystoid changes have long been described to occur in cases of serous macular and foveal detachment in the context of RRD, but they do not tend to occur so often in central serous chorioretinopathy (CSC) [29]. In a comparative study of eyes with RRD and eyes with CSC, Nakanishi et al. demonstrated the association of intraretinal cysts at baseline with a lower BCVA *after* resorption of similar amounts of subretinal fluid in both groups [29]. So far, available studies have found no clear consensus with regard to the prognostic factor of intraretinal cysts in RRD [11, 23]. But most importantly, no study is known to us which has quantified the amount of intraretinal fluid as suggested in this present study. We used the commonly accessible standardized ETDRS grid in order to distinguish between focal, none, and wide edema and found that wide cystoid changes were associated with significantly poorer functional outcomes, even after adjusting for the height of detachment, extent of detachment, surgical methods, and preoperative BCVA—other relevant factors that are commonly associated with a potentially poor outcome after CIRD [30]. Could extensive intraretinal edema ultimately reflect advanced, possibly irreversible photoreceptor loss as suggested by Nakanishi et al.? Our summarized findings, most notably the correlation of preoperative intraretinal edema with postoperative EZ integrity, seem to be in high accordance with this assumption.

## Limitations

There are several limitations to this study. Due to the inherent weakness of a retrospective analysis, assessment of exact duration and type of symptoms (e.g., mouches volantes vs. central vision loss) could not be assessed in a reliable manner. In our study, the duration of symptoms was not significant when correlated to the postoperative functional outcome, a finding which questions the reliability of this parameter in this study. Moreover, even though OCT was performed immediately before surgery in most cases included in this study, it cannot be excluded that the macular status of some patients may have changed in-between time of admission and time of surgery. Our results however do not seem to reflect this hypothesis to a strong degree, taking into account, in particular, the clear demarcation of BCVA values between patients with G3 and G4. Furthermore, evaluation for metamorphopsia was also not possible in this study, even though it is known as an important functional parameter, especially in eyes with foveal detachment [26]. The limited follow-up of 3 months postsurgery may also be considered as a limitation—a time span which admittedly cannot provide conclusive information about the final functional result. On the other hand, it can foreshadow a trend of functional recovery at a point of time, when possible cataract formation and redetachments (e.g., PVR-related) might still be of minor clinical relevance. At last, the variety of different surgical methods should be seen as a limitation to our study, even though there was no indication that this led to any statistical bias, especially because the relationship between the choice of more extensive types of surgeries (e.g., vitrectomy with encircling band) and lower visual outcomes seems quite comprehensible, given that extensive clinical and morphological findings may originally have led to this therapeutic approach. However, there was an indication that the type of surgery had an increased effect on postoperative BCVA (increased *B* values) in the multivariable model (compared to univariate regression), but the validity of this finding should be interpreted cautiously, especially since the size of surgery subgroups differed for both types of analysis, possibly leading to a limited comparability of raw coefficients in this case.

## Conclusions

This study may serve as a proposal for a classification of the macular status in patients with macula involving retinal detachment and illustrates the interplay and prognostic role of OCT biomarkers in the presence of center involvement. Most notably, the grade of detachment and the extent of intraretinal cystoid edema seem to represent good preoperative OCT biomarkers to predict functional recovery in cases with a detached foveal status. With our results, we would like to support a reassessment of morphological, clinical, and (peri-)operative factors in a standardized and comprehensible manner. Prospectively, multicentered studies should be conducted to further evaluate the clinical and scientific advantage of a coherent and reproducible description of the foveal status. This could not only be of high prognostic relevance but also contribute to already available guidelines by introducing a more objective and morphology-based assessment of the patient’s individual risk for long-term vision-loss, thus ultimately guiding the vitreoretinal surgeon in terms of immediacy and overall decision-making: when and how to surgically reattach the retina.

## Abbreviations

MIRD: Macula involving retinal detachment
CIRD: Center involving retinal detachment
RRD: Rhegmatogenous retinal detachment
TUM: Technical University Munich
IE: Intraretinal edema
SF: Subretinal folds
G: Grade
FDH: Foveal detachment height
RPE: Retinal pigment epithelium
EZ: Ellipsoid zone
ELM: External limiting membrane
IZ: Interdigitation zone
HM: Hand movement
CF: Counting fingers
f: Female
m: Male
OCT: Optical coherence tomography
SD: Spectral domain
CSC: Chorioretinopathia centralis serosa
PVR: Proliferative vitreoretinopathy
